# The Role of the Foreign Sector in the Spanish Bioeconomy: Two Approaches Based on SAM Linear Models

**DOI:** 10.3390/ijerph17249381

**Published:** 2020-12-15

**Authors:** Valeria Ferreira, Laia Pié, Antonio Terceño

**Affiliations:** Markets and Financial Analysis Research Group, Department of Business Management, Faculty of Business and Economics, Universitat Rovira i Virgili, 43204 Reus, Spain; valeria.ferreira@urv.cat (V.F.); antonio.terceno@urv.cat (A.T.)

**Keywords:** bioeconomy, linear multipliers, foreign sector, social accounting matrix, Spain

## Abstract

The bioeconomy emerges as an opportunity to focus on a more sustainable economy, avoid dependence on non-renewable resources and help to mitigate negative environmental impacts. The bioeconomy is considered a priority for the European Union and for Spain, which launched its strategy in 2016. To promote the Spanish bioeconomy, the impact and variables that may influence its development must be known. While previous works have analyzed the domestic sectors, this paper focuses on analyzing the economic importance of Spanish bioeconomy products and on the influence of and the existing links between the foreign sector and the rest of the economy. For this purpose, we apply two different methodologies based on linear social accounting matrix (SAM) models. The linkages show that many bioeconomy products have the potential to promote the rest of the economy and the values of some product multipliers become more significant due to their import dependence. These results enable us to know the structure of the Spanish bioeconomy and the relationships within its account. This analysis is a useful tool for developing policies focused on fostering the bioeconomy and economic growth.

## 1. Introduction

The world is currently facing major problems related to the environment that impact climate change, such as resource scarcity, excess waste generation and an increase in greenhouse gas (GHG) emissions [1]. In the 2030 Agenda published in 2015, the United Nations established seventeen goals for sustainable development that must be addressed by all member countries, the main purposes of which are to end poverty, protect the planet, and improve everyone’s lives and prospects [2,3].

Throughout 2020, political reaction focused on these issues has intensified in the European Union (EU). There has been the launch of the “European Green Deal” together with the “Just Transition Mechanism” and the “European Climate Law”, the main priorities of which are fighting climate change and making Europe climate-neutral by 2050 [4]. Moreover, climate change has a significant effect on health emergencies around the world [5], and the EU has created an economic recovery fund in response to the crisis caused by the COVID-19 pandemic, stating that this recovery should focus precisely on the environmental transition, aiming for a sustainable economy.

The environmental issues, together with the expected growth of the global population and our current lifestyles, demonstrate the need to look for alternative solutions focused on a sustainable path. Within this context, the debate emerges about the current economic model that must be reconsidered, focused mainly on environmental protection. Thus, the bioeconomy is considered as a way to mitigate part of these problems and the European Union (2012, 2018) and also many countries like Finland (2014), Spain and Norway (2016), France and Latvia (2017), Netherlands, United Kingdom and Ireland (2018), Austria and Italy (2019) and Germany (2020), have led the search for bioeconomy strategies [6,7].

The bioeconomy includes all the economic activities that cover the production and use of renewable biological resources and the conversion of these resources and their waste into value-added products such as food, feed, biological products, and bioenergy. Although it includes traditional activities such as agriculture, the “modern” bioeconomy also encompasses the production of other bio-based products such as biochemicals. The bioeconomy offers the possibility of finding new ways of supplying the same products based on the more efficient use of resources, reducing dependence on non-renewable resources, and avoiding resource depletion [8]. This impacts the creation of new products, increasing companies’ competitiveness and the development of new economic activities, thereby generating new jobs [7,9]. From a European perspective, a sustainable and circular bioeconomy is key to achieving the Green Deal’s ambition of making Europe the first climate-neutral continent by 2050 and for the economic crisis recovery caused by the COVID-19 pandemic focus on the environmental transition.

Despite its advantages, some issues are still discussed within the bioeconomy regarding the environmental perspective. It is important to promote the bioeconomy while avoiding causing environmental problems such as deforestation, increased GHG emissions, and food security. Therefore, these issues must be mentioned. The most recent debate is about the use of feedstocks (crops such as soybeans, corn, and sugar cane) to produce new bio-based products that are also used for food consumption, resulting in a competition between non-food bio-based and food products for them, and therefore in price increases and food safety risks [10]. Moreover, these types of crops in demand to produce biofuel are associated with indirect and direct land-use change, for example, the burning of forest areas, the marginal use of land caused by monoculture and the loss of biodiversity, among others [11,12]. The scientific literature focused on these disadvantages is still missing due to the lack of suitable environment variables data. However, there are some studies focused, for example, on the analysis of the footprint generated by the increasing consumption of biomass as input for bioproducts, in addition to the land-use change associated with them [13,14].

At the time of analyzing the development of the bioeconomy, the main problem is the lack of available data for bio-based products. For this reason, many studies have published different ways to quantify the bioeconomy contribution in an economy in the last recent years [15,16]. As an example, the bioeconomy social accounting matrix BioSAM), published for the European Commission for each member of the European Union, is a useful official database for the analysis of the bioeconomy. This matrix includes a detailed breakdown of the bioeconomy accounts and improved disaggregation of the non-agricultural bio-based accounts, such as bioenergy, biofuels, and bioindustries [17]. For the analysis of the Spanish bioeconomy in this paper, the BioSAM for Spain 2010 was converted into a symmetric bioeconomy matrix value at basic prices with a product-by-product framework, helping to describe and understand the Spanish bioeconomy structure and its relationships within its account in a more comprehensive way.

A social accounting matrix (SAM) is a database that enables us to represent economic and social information on all the transactions carried out among the agents of a specific economy during a period of time, and thus to determine how to obtain income and how to generate their expenditure [18]. They are considered to be very useful instruments to know the structure of an economy since they include the relationships between economic agents in terms of production, trade, income generation, consumption, saving, and investment. With this database, linear SAM models can be applied to analyze the capacity the different agents within an economy must generate and absorb the increments resulting from an exogenous injection of income in some of the accounts.

Accounts defined as exogenous are traditionally the productive factors and private activities and consumption, while endogenous accounts are the government, the savings-investment account, and the exterior sector [19,20]. However, these can be redefined according to the criteria of the researcher and depending on what is to be analyzed. For example, when the exterior sector is considered exogenous, the analysis of its effect becomes blurred, confounded by the impact generated by any of the other exogenous variables [18]. Therefore, the endogenization of the exterior sector is one of the possible modifications normally applied to traditional linear multiplier models, enabling the model to be extended to incorporate existing relationships between the economy and the exterior sector in the process of generating incomes. However, their application is not entirely straightforward, which is why, in the literature, different methodologies have been developed that enable the analysis of the effects of the exterior sector to be analyzed.

Multisectoral models have already been used for the analysis of the bioeconomy, for example, in Turkey [21], México [22], European Union 2000 [23,24], and 2007 [25], or Spain [26,27,28]. Moreover, the influence of the linear SAM models used for the analysis of an economy including the effects produced by the foreign sector is demonstrated in publications that have been produced in recent years, for Spain 2000 [29,30,31], and 2008 [18], or at the regional level for Cataluña [32,33,34] and Extremadura [35]. Nonetheless, no research on the influence of the exterior sector on the accounts that make up the bioeconomy has so far been identified.

The main objective of this article is to be able to analyze the economic importance of Spanish bioeconomy products and the links between them and the rest of the economy, as well as their influence on the foreign sector. With this aim in mind, the multisectoral linear SAM models have been used, and especially two methodologies that allow to better analyze the influence the exterior sector has on the rest of the economy.

This study is considered a first approach to knowing the influence of the foreign sector and to identifying the accounts of the bioeconomy that make the greatest contribution to generating income within the economy, the importance of international trade for each product, and in turn the dependence of some accounts on the foreign sector [18]. In this case, the analysis of the bioeconomy in Spain (2010) and the impact of the foreign sector on it using a new database specifically for bio-based products is an important contribution.

By including the foreign sector among the endogenous accounts, the role it plays in the process of income generation within the economy can be known. The results of this paper will enable us to know the influence that each of the accounts in the bioeconomy has in terms of diffusing or absorbing income, also considering the influence of the exterior sector and determining the accounts that are most dependent on it. The multiplier analysis is key to know the most important accounts in the bioeconomy in Spain and their impacts on economic growth. This affords the possibility of analyzing the results relating to their multiplier effect in greater depth, leading to a better understanding of the bioeconomy in Spain. The results will provide relevant information on the potential of the bioeconomy and its products and the foreign sector, which will be useful to promote its future development.

If the aim is to reduce the negative impact on the environment and to mitigate the causes of climate change, the analysis of the bioeconomy is a useful tool to provide key information essential for the development of socioeconomic policies focused on the promotion of the bioeconomy sectors with future potential in Spain.

In the next section, this article explains the SAM linear model and the different methodologies to analyze the foreign sector. Then, each model is applied for the case of the bioeconomy in Spain. The last part includes the discussion of the results and, later, the conclusions.

## 2. Materials and Methods

### 2.1. Database: Symmetric Bioeconomy SAM for Spain 2010

For this analysis, the original BioSAM matrix for Spain, published by the Joint Research Center of the European Commission, was converted into a symmetric bioeconomy SAM value at basic prices with a product-by-product framework, using model B of the Eurostat Manual of Supply, Use and Input-Output Tables [36]. A symmetric matrix has the advantage of being easy to handle and suitable for macroeconomic analyses [36,37]. A product-byproduct matrix describes the quantities of products used to produce each product, regardless of the sector that generates them [38].

The basic structure of the bioeconomy SAM symmetric product-byproduct is represented in Table 1. In this matrix, the rows represent the origin of the income from the different accounts, and the columns represent the expenditure or payments made, both in monetary terms. Therefore, the value in each cell (i, j) would represent a transaction between agent i and j, in which agent i receives an income from agent j for the sale of its products, used by j as intermediate consumption or final demand. This means that each cell simultaneously represents the payment from the account in the column to the account in the row.

Its interpretation indicates that the production system uses inputs to produce and then generates income from the intermediate or final sale of the production. To produce, in addition to intermediate consumption and imports, it is necessary to remunerate the factors of production, which represent income for the institutional sectors (such as household wages). This income will be spent in the productive sectors as consumption, transferred to other institutions and saved. Their consumption results in an increase in production and a continuation of the cycle [39].

The symmetric bioeconomy SAM contains 36 products in their aggregate form, 32 of which form part of the bioeconomy and 4 are unrelated to it. Furthermore, it includes an employment account, a capital account, two private accounts like companies and households, the government with the breakdown of net taxes (production, consumption, and direct taxes), savings and investment, and the rest of the world (Table 2).

### 2.2. The Foreign Sector in the Bioeconomy SAM

**Figure 1:**
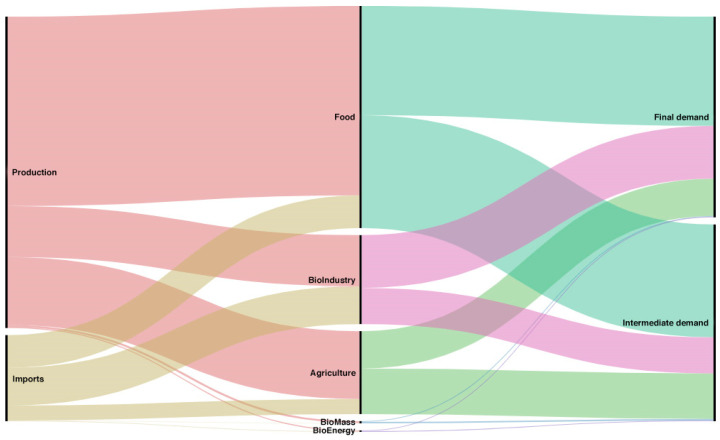
Product flows from production and imports to each account thereof. Source: own elaboration with Rawgraphs [40].

**Figure 2:**
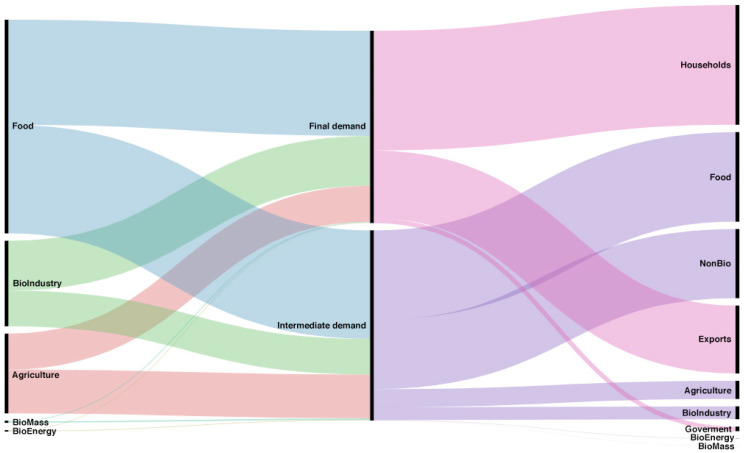
Product flows at intermediate demand and final demand and each account thereof. Source: own elaboration with Rawgraphs [40].

When formulating policies that promote the sectors of the bioeconomy, the analysis of the flow of the supply and demand of its products must include the influence the exterior sector has on both the imports and exports of each product. Figure 1 and Figure 2 provide a general overview of the role imports and exports play in the supply and the demand, respectively.

Figure 1 details the flow of the total supply of the bioeconomy. On the left-hand side of the Sankey diagram is the total supply of the bioeconomy from production and imports, and in the middle is the supply corresponding to each aggregated sector. On the right-hand side, we can see the total for supply destination, divided between final demand and intermediate consumption. The flow diagram clearly shows the importance of imports in the food, bioindustry, and agriculture sectors. A flow of imports towards the bioenergy sector is also observed, but with less significant values. There is a higher flow of the bioindustry sector towards final demand than towards intermediate demand.

Figure 2 allows us to visualize in a general way the destination of the bioeconomy products’ supply of the bioeconomy. On the left-hand side of the Sankey diagram is the total supply for each aggregated sector, and in the middle is the destination of the supply divided between final demand in household consumption, government, and exports, and intermediate consumption in other sectors. On the right-hand side, we can see the corresponding account of each demand. The food sector stands out, with its total offer directed mainly towards intermediate demand, but with high values directed towards final demand, and mainly households. Regarding the bioindustry, a large flow towards final demand, destined mainly for households and exports, is clearly observed. A similar trend towards both demands, although with significantly lower values, can be observed in the case of bioenergy, with the flow towards exports standing out in the final demand.

The analysis of the flows of supply and demand of bioeconomy products in Spain is shown in total values. We can therefore highlight the sectors with higher values, while those whose values are insignificant even though they represent a large percentage, for example of the exterior sector, do not stand out. Furthermore, this type of visualization does not enable the existing linkages between them to be analyzed.

Therefore, in the next section, the possibility of analyzing the exterior sector likewise as an endogenous account, and consequently knowing its importance and the interpretation of its links with the rest of the economy, is explained.

### 2.3. Multipliers Based on the Linear SAM Model

Using the linear SAM models is possible to analyze the information provided by a SAM regarding the structure of an economy. The origins of this methodology are found in the pioneering works of Stone [41] and Pyatt and Round [42]. They show the separate effects being generated in the economic activity of different agents due to the circular flow of income.

These models provide the opportunity to analyze the interlinkages among products, final demand and the distribution of income, and the sectors belonging to the bioeconomy and the rest of the economy. This type of analysis sheds light on the contribution of each of the bioeconomy products and enables us to identify those that are strategic in terms of generating wealth. These models are useful because they allow linear simulations to be made and the possible effects of applying measures to any of the exogenous accounts in the rest of the bioeconomy to be evaluated.

First, to be able to explain the model and know the effects of an exogenous injection on the economy through multipliers, the exogenous and endogenous accounts of the matrix must be defined. Exogenous accounts are determined outside the economic system and are the ones that can be used as potential economic policy instruments. According to Round and Pyatt [19] and Defourny and Thorbecke [20], the exogenous accounts are the government, savings–investment accounts, and the foreign sector. The endogenous accounts are the production sectors, factors (labor and capital), and private consumers [35]. This traditional assumption allows for capturing the complete relationship of the circular flow of income in a close economy [18]. The definition of the accounts can vary according to the criteria of the researcher and depending on what is to be analyzed. In order to analyze the foreign sector, the methodologies developed which enable the exterior sector to be included as an endogenous account, thus shedding light on how it influences the generation of income, need to be applied. These methodologies are explained in the following subsection.

The starting point of the linear SAM model is Leontief’s equilibrium equation but applied to the case of a SAM [43]. For more details, see Pié [44,45].

The standard representation is as follows:

(1) $$y_{n}{=A}_{\mathrm{nn}}y_{n}{+\;x}_{n}$$

(2) $$y_{n}{=(I\;-\;A}_{\mathrm{nn}}{)}^{-1}x_{n}{=M}_{\mathrm{nn}}x_{n}$$

where $y_{n}$ is the vector of endogenous income in each account, I is the identity matrix, $A_{\mathrm{nn}}$ is a matrix of structural coefficients (calculated by dividing the transactions in the SAM by the sum of the total column), where each element ${(a}_{ij})$ represents the proportion of total spending of the account j that spend on account i, and $x_{n}$ is a vector that represents the exogenous injections received by each endogenous account. ${\;M}_{\mathrm{nn}}={\left({I\;-\;A}_{\mathrm{nn}}\right)}^{-1}$ is the matrix of extended accounting multipliers of the SAM model, whose number of rows and columns is determined by the endogenous accounts used. This matrix shows the overall effects on the endogenous accounts of a unitary and exogenous change in the exogenous income of accounts. The sum of each element of the column $M_{\mathrm{nn}}$, shows the output multiplier (diffusion effect), $U_{.j\;}=\sum_{i=1}^{n}m_{\mathrm{ij}}\forall \;j=1,2,\ldots n,$ and the row sum is he multiplier of uniform expansion demand, representing the absorption effect $U_{i.\;}=\sum_{j=1}^{n}m_{\mathrm{ij}}\forall \;i=1,2,\ldots n$. Both of them are used to evaluate the capacity of each account to generate wealth in the rest of the economy.

The diffusion effect shows the income expansion effect generated in the endogenous accounts due to a unitary exogenous shock of income into the account. This effect is associated with backward expansion, which means that its input requirements to cope with the increase when it receives an exogenous injection are transferred to its suppliers [46]. This is explained by the fact that an exogenous injection that leads to an increase of one unit of final demand generates higher production needs. The linkages with the other accounts show that the increase in production is translated into a greater need for intermediate inputs, generating effects on the suppliers of these inputs and encouraging the development of the other activities. These inputs refer to the accounts considered as endogenous in the model, including products, labor and capital, and in part of this article also imports. This means that this effect makes it possible to see where the inputs used in production come from and the impact that an increase in production would have on the demand of the sectors from which these inputs will be bought [46]. A high value of this multiplier represents an account with a considerable backward income expansion influence on the rest of the economy due to the impact of one unit on the final demand of the sector analyzed [47]. The exogenous injection can be induced for an economic policy or another external event.

The absorption effect quantifies the increase in income in a sector as a result of a unit-income exogenous injection in the economy. It can be understood as a measure of forward-expansion because it indicates the relationship with its customers due to the distribution of its products in the rest of the economy. A unitary increase in each sector of the economy impacts the product analyzed if it is widely used by other sectors for their production. That distribution is related to intermediate demand between sectors and final demand that includes the private sector, and in the first methodology of this article, the exports are also considered as endogenous. The value obtained in the sum of the row shows how much this sector must increase its output due to an increase in one exogenous monetary unit in final demand over all the other endogenous accounts. It, therefore, indicates how much of the income growth that occurs is absorbed by each of the accounts. Thus, a high value of this multiplier has a significant impact on the other accounts in the economy, absorbing most of the global increase in income. That represents an account with a large forward measure of the chain of effects that involves the effect of the largest productions on the purchase of the client sectors.

#### 2.3.1. Analysis of the Foreign Sector in Linear SAM Models

When analyzing a country’s economy, it is important to know the influence of the foreign sector on it. If the linear SAM model is applied, including the foreign sector as endogenous, the interactions between the national economy and the rest of the world may not be correctly represented. The approach of this model implies that any increase in imports affects the entire economy, also impacting exports and generating an endlessly iterative process. Consequently, the obtained multipliers include the feedback effect of the linkages between imports and exports, and the role of the foreign sector in the income generation process, represented by the multiplier, may be overvalued [33].

Based on these limitations, two methodologies have been identified in the literature as possible solutions for the analysis and quantification of foreign sector influence on the rest of the economy, avoiding overvaluation [31]. One of them is based on endogenizing the foreign sector and analyzing its effects by decomposing the multipliers, and the other includes only the imports as endogenous. The two methodologies are explained in detail in the following points.

##### Multiplier Decomposition for the Analysis of the Foreign Sector

First, starting with the linear SAM model (Equation (2)), the multiplier matrix $\ddot{M}$ is obtained, including the foreign sector (imports and exports) as an endogenous account. If we defined n as the traditional number of endogenous accounts in a SAM, the new matrix would be of order (n+1×n+1). These cause overvaluation of the foreign sector in internal income and, therefore, a highly unreal multiplier [18,31,33]. For this reason, the multiplier decomposition technique is then applied to separate the feedback effect generated by the linkages between imports and exports.

In this case, starting with matrix $\ddot{A}$, the order (n+1×n+1), the matrix is then divided into two matrices,${\;A}_{1}$ and $A_{2\;}.$$A_{1}$, the order (n+1×n+1), including in $A_{11}$, $A_{12}$, $A_{21}$, is obtained with products or activities accounts, private consumption and zero values in the row and column of the foreign sector (The productive factors (labor and capital) are not considered in the decomposition of the methodology so that it can be represented more easily. As the authors indicate, the analysis presented does not change when these accounts are considered. Therefore, in the empirical application of this methodology, matrix $A_{1}$ will contain the values of the products, productive factors, and private consumption.). On the other hand, matrix $A_{2\;},$ the order (n+1×n+1) only includes the foreign sector account, the rest being zero, with $A_{31}$ as imports, $A_{13}$ are exports and $A_{23}$ private income from abroad.

$$\ddot{A}=\left[\begin{array}{ccc} A_{11} & A_{12} & A_{13}\\ A_{21} & 0 & A_{23}\\ A_{31} & 0 & 0 \end{array}\right]\Rightarrow {\;A}_{1}=\left[\begin{array}{ccc} A_{11} & A_{12} & 0\\ A_{21} & 0 & 0\\ 0 & 0 & 0 \end{array}\right]{\;A}_{2}=\left[\begin{array}{ccc} 0 & 0 & A_{13}\\ 0 & 0 & A_{23}\\ A_{31} & 0 & 0 \end{array}\right]$$

Therefore, starting with $\ddot{A}{=A}_{1}{+\;A}_{2\;}$, the calculation of the multiplier matrix is applied $\ddot{M}={\left(I-\ddot{A}\right)}^{-1}$, obtaining:

(3) $$\ddot{M}={\left({I\;-\;A}_{1}\;{-\;A}_{2}\right)}^{-1}$$

(4) $$\ddot{M}={\left[{\left({I\;-\;A}_{1}{)(I\;-\;(I\;-\;A}_{1}\right)}^{-1}A_{2}\right]}^{-1}$$

(5) $$\ddot{M}={\left[I\;-\;{\left({I\;-\;A}_{1}\right)}^{-1}A_{2}\;\right]}^{-1}{{(I\;-\;A}_{1})}^{-1}$$

where ${B=(I\;-\;A}_{1}{)}^{-1}A_{2}$, order (n+1×n+1) and making the following transformation:

(6) $${\left(I\;-\;B\right)}^{-1}={\left(I\;-\;B\right)}^{-1}{\left(I\;+\;B\right)}^{-1}\left(I\;+\;B\right)={\left({I\;-\;B}^{2}\right)}^{-1}\left(I\;+\;B\right)$$

The matrix of accounting multipliers $\ddot{M}$ can be defined by three multiplicative components, equal to:

(7) $$\ddot{M}={\left({I\;-\;B}^{2}\right)}^{-1}\left(I\;+\;B\right){\left({I\;-\;A}_{1}\right)}^{-1}={\ddot{M}}_{3}{\ddot{M}}_{2}{\ddot{M}}_{1}$$

where: ${\ddot{M}}_{1}={\left({I\;-\;A}_{1}\right)}^{-1}$ ${\ddot{M}}_{2}=\left(I\;+\;B\right)$ ${\ddot{M}}_{3}={\left({I\;-\;B}^{2}\right)}^{-1}$.

${\ddot{M}}_{1}{}_{\mathrm{nn}}$, has the following structure:

$${\ddot{M}}_{1}{}_{\mathrm{nn}}=\left[\begin{array}{ccc} \alpha_{1} & \alpha_{1}A_{12} & 0\\ A_{21}\alpha_{1} & \alpha_{2} & 0\\ 0 & 0 & I \end{array}\right]$$

where $\alpha_{1}={\left[I\;-\;\left(A_{11}{\;+\;A}_{12}A_{21}\right)\right]}^{-1}$ and $\alpha_{2}={\left[{I\;-\;A}_{21}{{(I\;-\;A}_{11})}^{-1}A_{12}\right]}^{-1}$

${\ddot{M}}_{1}$ is the own effects matrix. It reflects the effects caused to the economy due to the need to satisfy the new exogenous demand, which, due to the existing interactions between the accounts, causes increases in production, productive factors, and consumption. This matrix includes the income circular effect without considering the foreign sector. Therefore, its values coincide with the total effect of the traditional multipliers, according to Pyatt and Round [42].

${\ddot{M}}_{2}{}_{\mathrm{nn}}$ contains the following components:

$${\ddot{M}}_{2}{}_{\mathrm{nn}}=\left[\begin{array}{ccc} I & 0 & \alpha_{1}{(A}_{13}{\;+\;A}_{12}A_{23})\\ 0 & I & A_{21}\alpha_{1}A_{13}{\;+\;\alpha }_{2}A_{23}\\ A_{31} & 0 & I \end{array}\right]$$

${\ddot{M}}_{2}$ is the matrix of open effects between internal income and the foreign sector. This matrix represents the immediate effect (“first-round”) generated by the foreign sector [33]. Broadening the circular flow of income into an open economy includes the direct effect originating in the contribution made by the exterior sector to the income generation system in the economy [18]. That includes the effects caused to the rest of the accounts (production and consumers’ income) due to a unit increase in income received by the foreign sector accounts, and the impact on the import demand due to an exogenous income increase directed at the production sectors.

${\ddot{M}}_{3}{}_{\mathrm{nn}}$ has the following structure:

$${\ddot{M}}_{3}{}_{\mathrm{nn}}=\left[\begin{array}{ccc} \beta_{1} & 0 & 0\\ {(A}_{21}\alpha_{1}A_{13}{\;+\;\alpha }_{2}A_{23}{)A}_{31}\beta_{1} & I & 0\\ 0 & 0 & \beta_{2} \end{array}\right]$$

where $\beta_{1}={\left[{I\;-\;\alpha }_{1}{(A}_{13\;}{+\;A}_{12}A_{23}{)A}_{31}\right]}^{-1}$ and $\beta_{2}={\left[{I\;-\;A}_{31}\alpha_{1}{(A}_{13}{\;+\;A}_{12}A_{23})\right]}^{-1}$.

${\ddot{M}}_{3}$ is the matrix of circular effects, which includes the effects generated due to the circular flow of income. It reflects the feedback effect associated with the foreign sector, which implies that an increase in production is going to generate an increase in imports, which in turn generates a new round of effects and causes an automatic increase in exports. This imports-exports linkage may overvalue the interactions between the national economy and the foreign sector.

The additive decomposition equation is detailed for a better interpretation:

(8) $$\ddot{M}=\;{\ddot{M}}_{3}{\ddot{M}}_{2}{\ddot{M}}_{1}=\;I\;+\;\left({\ddot{M}}_{1}\;-\;I\right)\;+\;\left({\ddot{M}}_{2}\;-\;I\right){\ddot{M}}_{1}\;+\;({\ddot{M}}_{3}\;-\;I){\ddot{M}}_{2}{\ddot{M}}_{1}$$

(9) $$\ddot{M}\;-\;I=\;{\ddot{N}}_{1}\;+{\ddot{\;N}}_{2}\;+\;{\ddot{N}}_{3}$$

Equation (9) represents the multiplier net of the initial exogenous inflow that activate the multiplier process. Finally,$\;{\ddot{N}}_{1}$ is the own net effects,$\;{\ddot{N}}_{2}$ the net open effects and ${\ddot{N}}_{3}$ the net circular effects.

To avoid overvaluation of matrix $\ddot{M}$, the analysis of the foreign sector using the multiplier decomposition methodology focuses exclusively on ${\ddot{N}}_{1}$ and ${\ddot{N}}_{2}$. The analysis of these effects enables us to identify the internal economy’s own effects and the contribution of the foreign sector [31].

##### Endogenization of the Effect of Imports for the Analysis of the Foreign Sector

This alternative used for the analysis of the foreign sector is based on the inclusion of imports as an endogenous account and only the kept exports as an exogenous account [30,31]. This methodology allows for analyzing the real influence of the foreign sector. Using this, it is possible to avoid the overvaluation caused by the feedback effect that takes place when the exterior sector is considered as endogenous, which implies that the impact of an increase in imports is an increase in exports [30,31]. Hence, this methodology keeps to the assumption that a small country has no influence on decisions relating to the foreign sector [30,31].

This methodology implies a reformulation of the linear model. First, the dependence imports have on the total resources of a sector must be represented. According to Mainar et al. [30], within the linear models, the imports of sector i ($z_{i}$) can be expressed by a coefficient ${(h}_{i})$ which considers its value and the total output of the sector net of imports $y_{i}^{n}$.

Thus:

(10) hi=ziyin

Regarding the vector of coefficients $m^{*}=\;\left\{h_{i}\right\}$, this indicates the extent to which the resources of a sector come from the exterior sector [31]. Consequently, high values of these coefficients mean that an increase in the sector due to an exogenous demand shock will impact by increasing the demand for imports.

To apply this methodology in a social accounting matrix, the calculation of the matrix of coefficients $A_{\mathrm{nn}}$ needs to be modified. The new matrix $A_{\mathrm{nn}}{}^{*}$ is obtained considering the total output of each sector net of imports. Therefore, each element $a_{\mathrm{ij}}$ of the new matrix shows the expenditure of the i account for each monetary unit total expenditure of the account j, but net of imports.

Therefore, the linear model can be stated as:

(11) $${\;y}_{n}{}^{*}{=A}_{\mathrm{nn}}{}^{*}y_{n}{}^{*}{\;+\;x}_{n}{}^{*}{-\;z}^{*}{}_{n}$$

(12) $$y_{n}{}^{*}{=A}_{\mathrm{nn}}{}^{*}y_{n}{}^{*}{+\;x}_{n}{}^{*}{-\;H}^{*}{}_{\mathrm{nn}}{\;y}_{n}{}^{*}\;\Leftrightarrow {\;y}_{n}{}^{*}{=(I\;-\;A}_{\mathrm{nn}}{}^{*}{+\;H}^{*}{}_{\mathrm{nn}}{)}^{-1}x_{n}{}^{*}$$

Represented by,

$y_{n}{}^{*}\;n\times 1$ order is the vector of total resources net of imports.

$A_{\mathrm{nn}}{}^{*}\;n\times n$ order is the matrix of coefficients of endogenous variables calculated considering $y_{n}{}^{*}$

$x_{n}{}^{*}n\times 1$ order is the vector of exogenous variables that includes exports

$z^{*}{}_{n}$n×1 order is the vector of imports

$H^{*}{}_{\mathrm{nn}}$n×n order is the diagonal matrix with elements $m_{i\;}$(coefficients of imports).

With this methodology, a new version of the Leontief matrix is obtained (Equation (12)), ${\;(I\;-\;A}_{\mathrm{nn}}{}^{*}{+H}^{*}{}_{\mathrm{nn}}{\;)}^{-1}$, which enables the analysis of the impact generated on the income of endogenous account i, caused by an exogenous income unit injection in the endogenous account j, but discounting the influence of the external sector by the imports needed in the production process and subsequent income generation. Therefore, this alternative method allows the introduction of the external sector as an endogenous variable in the analysis with the linear SAM model, avoiding the effect of overvaluation in the multipliers. As a consequence, a sector whose influence of imports is important will reflect, as a result, a small multiplier value with this alternative.

## 3. Empirical Application for the Spanish Bioeconomy

This section includes the application of the two methodologies explained before to analyze the foreign sector in the Spanish bioeconomy matrix for 2010. The main objective is to know the influence of the foreign sector on Spanish income generation, specifically for the bioeconomy accounts. For this reason, we will compare the diffusion and absorption effects calculated after considering the foreign sector as endogenous.

With the decomposition methodology, it is possible to eliminate the feedback effect of the foreign sector and analyze the contribution of the foreign sector to the income generation system. This methodology allows the analysis of imports through the diffusion effect and the influence of exports through the absorption effect.

With the alternative methodology, the idea is to analyze the impact of the foreign sector account considering the assumption of a small country, endogenizing the effects of imports and keeping exports as an endogenous account. This alternative methodology reformulates the usual linear model, allowing the analysis of the diffusion effect and the influence of imports. This model discounts the values of multiplier expansion as a consequence of exogenous demand shocks, which are directly associated with a high percentage through import demand.

This implies that both methodologies allow us to appreciate the influence that the external sector has on the multiplier, either by including its impact within it or by discounting it. As a consequence, a sector whose influence of imports is important will reflect as a result of a higher multiplier when applying the decomposition methodology and a small value in the alternative.

The application of the two methodologies allows the effects of the external sector to be quantified more precisely, checking the consistency of both methodologies and therefore presenting more reliable results.

### 3.1. Analysis of the Foreign Sector with Multiplier Decomposition

The application of the linear SAM model with an endogenous foreign sector starts with the coefficients matrix ${\ddot{A}}_{\left(n+1\times n+1\right)}$ to obtain a multipliers matrix ${\ddot{M}}_{\left(n+1\times n+1\right)}.$ This matrix has 41 endogenous accounts because it includes a generic account for the foreign agent. With this matrix, it is possible to analyze the effects on consumption and production when the endogenous accounts mentioned before received exogenous injections.

The latter is then decomposed into three effects of which only the own and open net effects $\left({\ddot{N}}_{1}\;\mathrm{and}\;{\ddot{N}}_{2}\right)$ will be considered for the analysis. In Table 3, the results obtained for all the products of the diffusion and absorption effect are shown in ${\ddot{N}}_{1\;}$ and ${\ddot{N}}_{2}$, representing the effects of the domestic economy and the induced contribution of the foreign sector, respectively [18]. ${\ddot{N}}_{1}$ reflect the effects of the circular flow of income with the traditional assumption of endogenous accounts because it includes the relationship between production, income and consumption. The results of ${\ddot{N}}_{2}$ enable us to identify the contribution of the foreign sector in the process of income generation within the economy. Includes the effects on production and private income caused by the inflows of income from the foreign agent and the impact on the foreign sector income (an increase on imports) caused by an exogenous inflow into the activities [33].

This implies that in the endogenization of the foreign sector, the value of ${\ddot{N}}_{2}$ always makes the multiplier higher. Nevertheless, its variation should be taken into account to know if it causes a significant change in the importance of the multiplier. For this reason, the last two columns of Table 3 enable us to compare the variation of the multiplier when the foreign sector is considered as endogenous, given that it shows the proportion represented by the multiplier with the new case of the foreign sector on the situation without this case, with the aim of being able to analyze the cases with the greatest influence on it.

The results indicate that the diffusion multipliers are higher than the absorption multipliers for most bioeconomy products. If the average values of ${\ddot{N}}_{1\;}\;\mathrm{and}\;{\ddot{N}}_{2}$ are compared, it can be seen that in terms of the diffusion effect, the influence of the exterior section is greater, while it is very low for the absorption effect. This can be clearly appreciated in the last two columns of Table 3.

**Figure 3:**
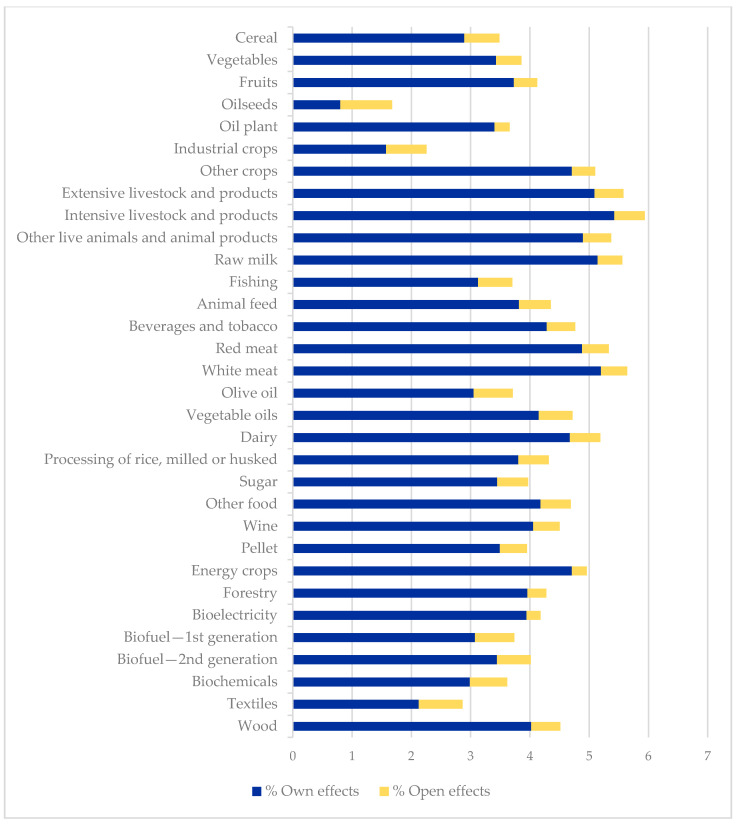
Diffusion multiplier effects: Own ${\ddot{N}}_{1}$ and open ${\ddot{N}}_{2}\;$ effects. Source: own elaboration.

Taking diffusion multipliers into account, representing in the first and second columns of value Table 3 and in Figure 3. The net diffusion effect represents how a unitary increase in the demand of the sector analyzed is transferred to the rest of the accounts. This means, for each monetary unit of income received by it, the multiplier will allow knowing how many monetary units of income are generated over the rest of the sectors considering the circular flow of income. This impact will include the direct, indirect and induced effects generated by the consumption of inputs from other sectors, the use of capital and labor, and in this case, the inclusion of imports necessary for the total supply of that product. For a better interpretation of the multiplier, it is analyzed separately ${\ddot{N}}_{1\;}$ and the influence of the foreign sector (${\ddot{N}}_{2}$).

Evaluating ${\ddot{N}}_{1\;}$, most of the accounts belonging to agriculture, biomass and food stand out, with an above-average diffusion multiplier. The accounts related to other crops, livestock, the meat industry, raw milk and dairy stand out with a multiplier higher than 5. This indicates that for each monetary unit of income received by these sectors, a total expansion of more than five monetary units is created over economic activity as a whole. Other products have a diffusion multiplier less than 5, but above-average, like other crops, animal feed, beverages and tobacco, red meat, vegetable oils, dairy, rice, other food products, wine, energy crops and forestry, stand out. Other bioeconomy accounts, including in bioenergy and the bioindustry (such as biofuels and biochemical), have a below-average income diffusion effect, but bioelectricity and wood stand out. The accounts with a low diffusion effect are cereal, oilseeds, industrial crops, biochemical, and textiles.

The contribution made by the exterior sector to the income generation system can be analyzed for the diffusion effect, considering ${\ddot{N}}_{2}$. The analysis demonstrates the maximum values for the bioeconomy products within the bioenergy (biofuels), bioindustry (biochemical and textiles), and agriculture (cereal, oilseed and industrial crops) groups. Outside the bioeconomy, natural resources and manufacturing accounts can also be highlighted. The lowest values are for services, bioelectricity, energy crops, forestry, and oil plants.

On comparing the two effects, what is highlighted is the fact that the products with non-significant own effect, such as biofuels, are mostly those that contain the highest open effect values. For the products mentioned, the value of the multiplier when considering the influence of the foreign sector increases by more than 0.6. These imply that the impact of an increase in production due to an exogenous increase in demand will be linked to an increase in other sectors and factors of productions, but mostly in their foreign sector.

The analysis of the percentages in column 5 of values of Table 3 quantifies by how much the own multiplier diffusion effect increases on taking the exterior sector into account, showing an average increase of approximately 17%. This increase emerges because the income creation process now includes the linkages there are between the income of the sectors and the demand for imports [33]. However, if this column is analyzed for each account, it can be observed that the above-mentioned accounts stand out with an increase in the multiplier effect of more than 20%, and in some cases more than 50%, on including the foreign sector.

The calculation of the diffusion effect highlights the importance of several products of the bioeconomy when demanding inputs from other products for their own production. The analysis shows that many of the bioeconomy’s products classified within its traditional sectors, such as agriculture, food and biomass, are suitable to be promoted through policies since they will have an above-average backward impact on the rest of the economy. This impact is represented by their consumption of other products, capital and labor, and to a lesser extent by imports. However, the analysis of the influence of the foreign sector allows us to observe that some products mentioned above, despite not having an above-average multiplier effect, the multiplier value increases when considering the influence that imports play in the total supply necessary to satisfy the total demand.

**Figure 4:**
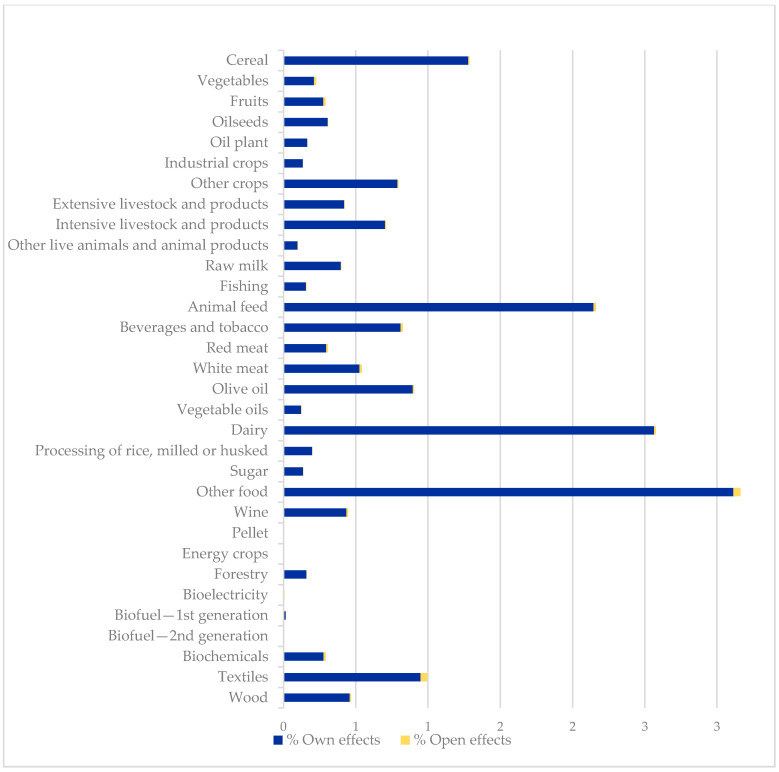
Absorption multiplier effects: Own ${\ddot{N}}_{1\;}$ and open ${\ddot{\;N}}_{2\;}\;$ effects. Source: own elaboration.

On evaluating the absorption effect (the third and four columns of values of Table 3, and Figure 4), each multiplier represents to what extent the production of a sector is used by other products in their production process, indicating the relationship with its customers, due to the distribution of its products in the rest of the economy. That distribution is related to intermediate demand between sectors and final demand that includes the private sector and also, in this case, the exports. This means that the values of this multiplier show how the products analyze need to increase their output due to an increase of a monetary unit of the final exogenous demand in each sector. For a better interpretation of the multiplier, it is analyzed separately ${\ddot{N}}_{1\;}$ and the influence of the foreign sector ${\ddot{N}}_{2}$.

${\ddot{N}}_{1\;}$ does not stand out for the bioeconomy sectors. The food and bioindustry groups have the highest absorption impact, and the accounts related to biomass and bioenergy have extremely low values. The analysis of the absorption effects shows that for both ${\ddot{N}}_{1}$ and ${\ddot{N}}_{2}$ the values are very low and are mainly important in the accounts classified as non-bioeconomy, with services and manufacturing standing out. When analyzing the absorption effect, we set aside the values of the accounts of the private institutions because they represent values that are far higher than the rest, given that they are recipients of all the resources of the capital and labor factors. If the average of the accounts of products is considered, the products within the bioeconomy animal feed, dairy and other food products stand out with a multiplier above-average. These products have a net absorption multiplier of more than two; this means that when a monetary unit is injected into the set of accounts, these products will increase their income levels by more than two monetary units.

The last column in the table shows the increase in the absorption effect when the effects of the foreign sector are included in the multiplier, compared to the traditional assumption. The total average of the absorption effect only increases by 2%, with biofuels, biochemicals, textiles, and vegetables and fruits mainly standing out within the bioeconomy, indicating that the exterior sector impacts more on these sectors, benefitting from exogenous injections in exports.

In general, the net values of this absorption effect show that the output of the bioeconomy products is not used by many accounts. This assumes that their products can be highly demanded by intermediate demand for other products or by private consumption and exports, but that this demand is concentrated in a few accounts. In spite of the low influence of forward linkages for biofuels, biochemicals, textiles, vegetables and fruits, due to the low intermediate demand for these products by the other sectors, this analysis allows us to identify that for these products, the demand from the foreign sector stands out.

### 3.2. Analysis of the Foreign Sector with Endogenization of Imports Effects

Continuing with the analysis of the foreign sector, the second methodology explained in Section “*Endogenization of the Effect of Imports for the Analysis of the Foreign Sector*”, which is mainly based on the endogenization of imports while maintaining exports as exogenous, is applied. The results are presented in Table 4, each column of which shows the values of the diffusion effect calculated using each methodology. This table enables the effects generated by a unit exogenous final demand shock in any of the endogenous accounts for each methodology to be easily compared.

Table 4 is separated into two parts. In the first part, the first column of values shows the diffusion multipliers on considering the foreign sector to be exogenous. The next two columns represent the different cases with the foreign sector as endogenous. The second column shows the total multipliers with the endogenization of the foreign sector (without applying any methodology to correct its value). The next column shows the results obtained by applying the alternative methodology of this section, considering only the imports as endogenous. The columns of values in the second part of Table 4 show the position of the analyzed accounts according to their multiplier for each of the above-mentioned methodologies. Attention should be paid to the variation in the multiplier value with the imports considered as endogenous and whether this leads to a change in its ranking among the accounts within the economy.

A comparison of the results obtained and shown in column 2 of the table with the results in column 1 clearly demonstrates the importance of applying a methodology that allows for analyzing the effects of endogenizing the foreign sector without taking the overvaluation of multipliers due to the feedback effect into account. The validity of the suggested method is thereby proven with the more real multiplier values represented in columns 3.

The results obtained with this alternative methodology show consistency in comparison with the decomposition methodology analyzed above. Using this alternative methodology, those products whose production comes mainly from abroad are more sensitive due to the elimination of the effects of imports. This can be seen in the case of products such as textiles, biofuels, biochemicals, cereals, oilseeds, olive oil and industrial crops, for which their multiplier effect is reduced to their domestic effects, which in these cases is very small and can be seen in column 3.

Moreover, Mainar et al. [30] and Fuentes et al. [31] mention that the multipliers calculated considering this methodology, where only the endogenization of imports is applied directly, enable more adjusted results to be obtained. This can also be clearly appreciated on considering the columns in the second part of Table 4, where the position of each account is detailed according to the multiplier value.

If we take the initial situation where the foreign sector is considered exogenous with the proposed methodology, it can be seen that similar distributions are obtained [31]. In addition, although ranking changes were identified in some of the accounts, they were not as abrupt as when the overvaluation of the multiplier (column 5) was not eliminated. This can clearly be seen with the olive oil and first-generation biofuels accounts, for example, where the uncorrected multiplier effect is so high that it leads to a better position in the total ranking.

## 4. Discussion

As mentioned in the results, the analysis of the multiplier effect allows us to know those products of the bioeconomy that have a greater impact on the rest of the economy and therefore, a change of one euro in the final demand of these sectors generates an increase in the activity of the other sectors higher than one euro. The results obtained show that for the bioeconomy products, the diffusion effect stands out. This means that several products of the bioeconomy have the capacity to generate wealth above-average for their input suppliers.

The net diffusion effect results show that among bioeconomy products, those grouped under food, biomass, and agriculture stand out for their greatest influence. According to Table 3, there are many bioeconomy products that stand out with a multiplier higher than 4. This indicates that for each monetary unit of income received by these sectors, a total expansion of more than four monetary units is created over economic activity as a whole. Clearly, these will be products of the bioeconomy that could be taken into account when implementing policies that seek to promote other sectors of the bioeconomy through monetary injection.

The novelty of the matrix used in this article is that it provides disaggregated products for the bioenergy and bioindustry groups. The diffusion effect within this group stands out only for bioelectricity due to the influence of capital, with values very close to the average in the case of biofuels. Regarding the bioindustry, only wood products have an above-average diffusion effect.

However, by endogenizing the effect of the foreign sector, the low multiplier of some products will be increased and become more significant due to their import dependence. This analysis enables us to explain the relevance of the exterior sector on the multiplier effect generated in the economy for each sector specifically. The results obtained show that the exterior sector is clearly influential as an element to increase the diffusion effect in some of the accounts of the bioeconomy. By including the analysis of the influence of the foreign sector, it is possible to observe that some products, despite not having an above-average multiplier effect, do increase when considering the influence that imports play in the total supply necessary to satisfy the total demand. This analysis shows that these are related to biofuels, biochemicals, textiles, oilseed and industrial crops, for example. This assumes that they are products whose dependence on the external sector is significant and that, therefore, any increase in their demand will include the necessary imports within the increase in inputs.

In this case, it can be seen that these are products whose Spanish production is not yet well developed, and the influence of the foreign sector shows by imports stand out in the total supply, mainly in agriculture at more than 40% for cereals, oilseeds, and industrial crops, and at more than 30% for biofuels, biochemicals, and textiles. Therefore, the linkages between the income of the sectors and the demand for imports show that when there is an increase in production due to an increase in demand, it carries with it an increase in the inputs necessary for its production and therefore in imports, so the impact will be linked to an increase in their foreign sector. The promotion of the bioeconomy in Spain can consider this information and promote policies that encourage local production or better import trade conditions.

Therefore, considering the alternative methodology applied, it can be appreciated that for certain bioeconomy accounts, it is commercial relations with the exterior that cause their expansive effects on the rest of the economy, since if only the multiplier is analyzed without endogenizing this sector, its values are low. The accounts whose production comes mainly from the exterior are more sensitive to changes in the multiplier when the effect of imports is included.

The high diffusion multiplier indicates that various inputs of other products are used for their own production, which in this case includes other products, labor, capital and imports, therefore if demand is stimulated, the impact on the rest of the economy will be above average.

According to the results, some products with the greatest influence on wealth shown by its multipliers are important to promote the bioeconomy in Spain and, by promoting their final demand, they can influence the rest of the economy. These are mainly the ones mentioned previously; however, to shift towards a Spanish bioeconomy, those sectors without significant values in the economy or those whose multipliers do not stand out should also be considered (mainly those included within bioenergy and the bioindustry).

The main objectives of the Spanish Bioeconomy Strategy Horizon 2030, published in 2016, focus on the agri-food sector, forestry, the bioindustry, and bioenergy, demonstrating that these goals must be achieved to create an economy that uses biological resources and avoids fossil-based resources. Consequently, the Spanish bioeconomy must also focus on promoting the sectors with future potential, supporting research and eco-innovation, encourage the application of technologies, capital investment and the promotion of bio-based products [7,48,49].

The transition from a fossil-based economy to a bio-based economy is already a reality. The priority over the last year has been funds provided for the economic recovery due to the COVID-19 crisis, with a special focus on promoting the transition to a sustainable economy. According to this, research on the bioeconomy and related topics need to be increased to know the impact of the pandemic in some sectors and to identify which ones are more appropriate to promote to achieve the required economic recovery in a more sustainable way. For this reason, this type of information is important, especially for policymakers in decision-making processes related to plans and investments whose objective is the promotion of the Spanish bioeconomy. This study is considered a first approximation to be able to know and quantify the importance and influence of the foreign sector in the generation of income of the economy, but it can be deepened with the inclusion of data that extend the detail of the study [18].

Although these results enable us to identify the most suitable sectors to be promoted for developing policies focused on fostering the bioeconomy and economic growth, this article also focuses on the economic analysis of the bioeconomy in Spain, without taking any social or environmental variables into account. As we mention in the introduction, there are some critical points regarding this issue that need more attention. This means that future lines of research could consider some variables for the environmental analysis of the bioeconomy based on objectives previously established by other similar policies. In this way, decisions that really consider a complete sustainable approach can be taken, as an example, environmental problems such as deforestation, increased GHG emissions, competing pressure for land use and food security. In this case, the multiregional input–output models enable the economy to be monitored not only the country of analysis and also disaggregated the account rest of the world, considering a variety of socioeconomic and environmental data [13,14,50,51].

## 5. Conclusions

In 2020, a significant step forward was taken at the European level to face the challenges of the environment and climate change. First, there was the “European Green Deal”, whose main objective is fighting against climate change and making Europe and each member country climate-neutral by 2050 [4]. To achieve this end, a transformation of the current lifestyle, work, production, and consumption patterns was proposed. Later the same year, and prompted by the economic crisis caused by the COVID-19 pandemic, the European Union created an economic recovery fund in which environmental transition is considered as one of the strategic objectives to which resources must be allocated. The funds are focused on Europe’s economic recovery while also taking climate change and a modern, clean, healthy economy focused on sustainability, guaranteeing the livelihood of future generations, into account. In terms of these funds, Spain is one of the countries that is benefitting most. To achieve these goals, the European Union stresses the importance of focusing on a circular bioeconomy.

The general objective of this article is to contribute to the knowledge of the bioeconomy in Spain. For this purpose, the economic structure and the interrelations between its accounts and the rest of the economy are analyzed to identify the possible impact of bioeconomy products in terms of generating economic growth. The aim of this paper is to apply different methodologies established with SAM matrices, allowing for a multisectoral analysis of the economy that includes the influence of the foreign sector to be carried out. This type of analysis is a useful tool to provide key information essential for policymakers in decision-making processes related to investment destinations policies focused on the promotion of the bioeconomy sectors with future potential in Spain together with reducing negative impacts on the environment.

The traditional model of linear multiplier considers the foreign sector as an exogenous account, avoid considering important information about the income generation sources for some products which have a high level of interaction with foreign markets. However, if we only include the foreign sector as an endogenous account, the model considers it within the circular flow of income, and the problem is that the national economy then influences the foreign sector, meaning that any increase in imports automatically leads to an increase in exports [33]. This leads to error and consequently to overestimated multipliers. This explains why the possibility of applying variants for the analysis of the exterior sector with these models is fundamental. For this reason, we apply two methodologies suitable to isolate the expansive effect caused by the foreign sector. Both of them attempt to know the influence of and the existing links between the foreign sector and the rest of the economy, and particularly the sectors belonging to the bioeconomy.

One of the main conclusions of this article is that many bioeconomy products have the potential to promote the rest of the economy, confirming that these are key bioeconomy products for receiving exogenous income injections. The bioeconomy in Spain is more focused on traditional sectors such as agriculture, biomass, and food. The more innovative bio-based products have not yet played a significant role in the production, having a great influence on imports.

Hence, this article concludes that to promote the Spanish bioeconomy, the sectors with the greatest influence on wealth, thus encouraging the economy and shown by its multipliers, must be stimulated. These are mainly the ones mentioned previously. These products—fruit, other crops, extensive and intensive livestock and products, and raw milk—are in the agriculture group, considering the biomass group (energy crops and forestry) and wood products to be within the bioindustry, and only white and red meat and rice within the food group. However, in the application of this analysis for the bioeconomy in Spain, considering the foreign sector generates differences if we only consider the traditional multiplier methods. The importance of the analysis of the influence of the exterior sector is especially evident in terms of the diffusion multiplier. On its inclusion, there is an increase in the multiplier focused mainly on the accounts within bioenergy, such as biofuels, the bioindustry, biochemicals, textiles, and oilseed and industrial crops within agriculture. Without taking the influence of the exterior sector into account, these accounts have a low multiplier effect, underlining the importance of this methodology when considering the dependence of the exterior sector in the analysis of each sector.

The conclusion of this paper is that many bioeconomy products have the potential to promote the rest of the economy, providing the opportunity to analyze possible policies that address the relevant accounts. Nevertheless, those without significant values in the economy or those whose multipliers do not stand out should also be considered. From this perspective, these sectors should also be promoted (mainly those included within bioenergy and the bioindustry) to shift towards a bio-based economy. The transition from a fossil-based economy to a bio-based economy is already a reality. Consequently, the Spanish bioeconomy must also focus on promoting the sectors with future potential.

The final conclusions of this article thereby provide the opportunity to understand the behavior of the bioeconomy in Spain and to evaluate possible measures that can be taken to promote its development. Consequently, this research suggests that the bioeconomy in Spain still has a long way to go. To progress, not only do the most prominent products need to be promoted, but some of the less important sectors need to be promoted too. In so doing, demand for their products and their production also increases, having a knock-on effect on the rest of the economy. For this to happen, these sectors should be boosted, encouraging investment, innovation, and research, enabling new ways of bio-based production to be obtained, with these new products promoted in the market to increase demand for their development. As their participation in the market increases, their production will likewise be increased, together with the use of inputs from other sectors and employment. The results suggest, for instance, that the use of some bioproducts could be promoted with policies offering an extra stimulus for using them. For example, there could be benefits for companies that use renewable energy and bio-based inputs or policies that set mandatory percentages for the consumption and sale of some bio-based products. The production of bio-based products is related to the promotion of biorefinery industries. Moreover, policies can intervene in the sectors whose production is largely distributed towards final demand by promoting the consumption of more sustainable bio-based products.

To this end, greater collaboration and research is required among stakeholders and interested parties, considering transdisciplinary multifactor work-schemes that helps to achieve the transition to a more competitive and sustainable economy [52]. In particular, it would be interesting to incorporate the skills and competencies related to sustainability in educational institutions and also training for employers, motivating and allowing them to lead a transformational change [53].

It is necessary to mention the caveats and limitations of this research. Regarding the database, the starting point in the construction of the bioeconomy SAM symmetric product-byproduct at basic prices was the BioSAM, the construction of which involves some difficulties and estimations [26] which were also encountered when it was converted to the matrix with the previously explained characteristics. Furthermore, although the matrix used was the last official database published in 2018, it also contains data for the year 2010. Considering that the bioeconomy is an issue that has emerged in recent years, and the Spanish strategy was promoted in 2016, the conclusions may not reflect the current structure of the economy and therefore of the bioeconomy in Spain. For this reason, we try to distinguish between those conclusions whose meaning can be taken as a variant by the passage of the last years and those in which it can be considered invariant.

In order to correctly conduct this discussion, we believe it is appropriate to consider, on one hand, the analysis of the latest Spanish Input–Output table published for 2016 in December 2019, and on the other hand, specific policies related to the bioeconomy that have been promoted and may influence it variation in recent years.

The analysis shows that, on one hand, the more traditional sectors of the bioeconomy, such as agriculture and the food industry, represent a similar trend in recent years in the economic structure of the Spanish economy. Mainly, agriculture has always been an outstanding sector in Spain, and its trend shows continuous growth.

On the other hand, considering the aggregated data in this table, and also the policies promoted in recent years, we can say that the conclusions on those modern bio-based sectors can be considered to vary more quickly over time. However, due to the lack of disaggregation of the bioeconomy sectors, we cannot know the influence of bioenergy and bioindustry products in recent years.

Considering that the Spanish Bioeconomy Strategy focuses on agri-food, forestry, the bioindustry, and bioenergy, we understand that the sector with the greatest variation in recent years could have been mainly bioindustry and bioenergy [9]. Possibly, the greatest variations could occur within the Bioenergy sector due to its low representation in our database and considering the biofuel policies published during the last decade both at the European and Spanish level. The latter, classified under the “Renewable Energy Directive”, has focused on promoting the use of biofuels, but at the same time, limiting the use of conventional biofuels obtained from feedstocks used for human or animal consumption. Moreover, the new regulation lays down that the use of crops for biofuel production requires sustainability certification, aimed at guaranteeing that the raw material does not come from protected areas with high biodiversity or biodiversity in danger of extinction. This change will produce a variation in the production of biofuels, and therefore in the consumption of raw materials and imports [54,55]. A clear example is the limitation of the use of palm oil to produce biodiesel, which is to be banned by 2030, and thus the increased use of other crops such as soya. Thus, the use of biofuels that come from sustainable crops and the increase in second- and third-generation biofuels is expected. This will have effects on the consumption of inputs and the production of bioproducts.

For these reasons, the construction of a matrix with updated bioeconomy data is necessary to be able to continue with this type of analysis and evaluate the application of the different policies.

As for the methodologies used, the limitations of the multisectoral linear SAM model mainly including the linear behavior of economic agents, constant technical coefficients over time, fixed prices, and excess capacity [20]. Meaning that both the structure of inputs and the amount from the foreign sector will be fixed values [33].

Despite the above-mentioned limitations, every methodology applied in this thesis has already been used in previous research, and they have all been cited as examples. Likewise, the results obtained must be interpreted with caution, considering the limitations of the model and the database.

## Figures and Tables

**Table 1:** Bioeconomy social accounting matrix (SAM) symmetric product-by-product basic structure for Spain.

	Products	Factors of Production	Private Sectors	Public Sectors	Saving and Investment	Rest of the World (RoW)	Total
**Products**	Intermediate consumption		Consumption private sector	Expenditure public sector	Investment and stock changes	Exports	Demand
**Factors of production**	Remuneration of factors					Factor income from RoW	Factor Income
**Private Sectors**		Factor income to the private sector	Transfers between the private sector	Transfers to the private sector		Transfers to the private sector	Private income
**Public Sectors**	Taxes	Factor income to public sector	Private sector taxes			Transfers to Government	Public income
**Saving and Investment**			Private sector Savings	Public sector Savings		Transfers from RoW	Saving
**Rest of the World**	Imports	Factor income to RoW	Transfers private sector to RoW	Transfers public sector to RoW			Payments to RoW
**Total**	Supply	Expenditure on factor	Private expenditure	Public expenditure	Investment	Income from RoW	

Source: own elaboration based on Mainar et al. [37].

**Table 2:** Description of accounts in the bioeconomy SAM for Spain.

Products		Other Accounts
**Agriculture**	**Food**	**Factors of Production**
Cereal	Sugar	Labor
Vegetables	Other food	Capital
Fruits	Wine	**Taxes**
Oilseeds	**Biomass**	Net production taxes
Oil plants	Pellet	Net products taxes
Industrial crops	Energy crops	Direct taxes
Other crops	Forestry	**Private and public agents**
Extensive livestock and products	**Bioindustry**	Households
Intensive livestock and products	Biochemicals	Enterprises
Other live animals and animal products	Textiles	Government
Raw milk	Wood	**Capital account**
Fishing	**Bioenergy**	Investment—savings
**Food**	Bioelectricity	**External relations**
Animal feed	Biofuel—1st generation	Rest of the World
Beverages and tobacco	Biofuel—2nd generation	
Red meat	**Non-bioeconomy**	
White meat	Natural resources	
Olive oil	Manufacture	
Vegetable oils	Energy	
Dairy	Service	
Processing of rice, milled or husked		

Source: own elaboration based on Mainar [17].

**Table 3:** Net diffusion and absorption multiplier effects (with the endogenous foreign sector).

		Net Diffusion Effect		Net Absorption Effect		% ${\ddot{N}}_{2}/{\ddot{N}}_{1}$	
Group	Product	${\ddot{N}}_{1}$	${\ddot{N}}_{2}$	${\ddot{N}}_{1}$	${\ddot{N}}_{2}$	Diffusion	Absorption
**Agriculture**	Cereal	2.894	0.595	1.280	0.006	21%	1%
	Vegetables	3.432	0.425	0.211	0.017	12%	8%
	Fruits	3.732	0.390	0.275	0.016	10%	6%
	Oilseeds	0.803	0.872	0.306	0.002	109%	1%
	Oil plants	3.406	0.255	0.165	0.001	7%	1%
	Industrial crops	1.574	0.683	0.134	0.001	43%	0%
	Other crops	4.710	0.394	0.789	0.006	8%	1%
	Extensive livestock and products	5.092	0.487	0.420	0.004	10%	1%
	Intensive livestock and products	5.423	0.517	0.702	0.008	10%	1%
	Other live animals and products	4.897	0.474	0.097	0.001	10%	1%
	Raw milk	5.144	0.417	0.398	0.002	8%	1%
	Fishing	3.126	0.578	0.156	0.004	18%	2%
**Food**	Animal feed	3.816	0.534	2.145	0.017	14%	1%
	Beverages and tobacco	4.285	0.478	0.811	0.015	11%	2%
	Red meat	4.880	0.451	0.296	0.011	9%	4%
	White meat	5.197	0.442	0.526	0.017	9%	3%
	Olive oil	3.052	0.662	0.895	0.008	22%	1%
	Vegetable oils	4.149	0.570	0.122	0.003	14%	2%
	Dairy	4.673	0.513	2.564	0.012	11%	0%
	Processing rice, milled or husked	3.804	0.513	0.198	0.001	13%	1%
	Sugar	3.451	0.518	0.137	0.001	15%	1%
	Other food	4.178	0.512	3.114	0.048	12%	2%
	Wine	4.054	0.449	0.436	0.011	11%	3%
**Biomass**	Pellet	3.495	0.457	0.000	0.000	13%	2%
	Energy crops	4.708	0.251	0.001	0.000	5%	1%
	Forestry	3.957	0.317	0.160	0.001	8%	1%
**Bioenergy**	Bioelectricity	3.944	0.235	0.005	0.000	6%	2%
	Biofuel—1st generation	3.074	0.663	0.015	0.001	22%	6%
	Biofuel—2nd generation	3.446	0.567	0.004	0.000	16%	6%
**Bioindustry**	Biochemicals	2.988	0.630	0.277	0.016	21%	6%
	Textiles	2.126	0.736	0.948	0.047	35%	5%
	Wood	4.020	0.492	0.458	0.009	12%	2%
**Non-bioeconomy**	Natural resources	1.370	0.852	3.063	0.105	62%	3%
	Energy	4.359	0.418	3.467	0.074	10%	2%
	Manufactures	3.078	0.620	9.172	0.586	20%	6%
	Services	4.587	0.308	31.620	0.993	7%	3%
	**Average (productive accounts)**	3.798	0.502	0.564	0.009	17%	2%

Source: own elaboration. Database: symmetric bioeconomy SAM for Spain 2010.

**Table 4:** Diffusion multiplier effect and position for each account according to the endogeneity criteria model of foreign sector used.

	Diffusion Multiplier Effect			Position of Each Product in the Ranking		
	Foreign Sector Exogenous	Foreign Sector Endogenous		Foreign Sector Exogenous	Foreign Sector Endogenous	
Product	${\left(I\;-\;A\right)}^{-1}$	${(I\;-\ddot{\;A})}^{-1}$	${\;(I\;-\;A}^{*}{+H}^{*}{)}^{-1}$	${\left(I\;-\;A\right)}^{-1}$	${(I\;-\;\ddot{A})}^{-1}$	${\;(I\;-\;A}^{*}{+H}^{*}{)}^{-1}$
Cereal	3.89	8.62	3.30	32	25	32
Vegetables	4.43	7.82	4.01	25	32	24
Fruits	4.73	7.83	4.34	21	31	19
Oilseeds	1.80	8.74	0.93	36	22	36
Oil plants	4.41	6.44	4.15	26	36	22
Industrial crops	2.57	8.00	1.89	34	30	34
Other crops	5.71	8.84	5.32	7	21	8
Extensive livestock	6.09	9.97	5.61	4	2	4
Intensive livestock	6.42	10.54	5.91	1	1	1
Other live animals	5.90	9.66	5.42	5	6	7
Raw milk	6.14	9.46	5.73	3	8	3
Fishing	4.13	8.73	3.55	27	23	27
Animal feed	4.82	9.07	4.28	19	14	21
Beverages and Tobacco	5.29	9.09	4.81	12	13	12
Red meat	5.88	9.47	5.43	6	7	6
White meat	6.20	9.72	5.75	2	4	2
Olive oil	4.05	9.31	3.39	30	10	30
Vegetable oils	5.15	9.69	4.58	14	5	17
Dairy	5.67	9.76	5.16	9	3	10
Processing rice	4.80	8.89	4.29	20	20	20
Sugar	4.45	8.57	3.93	23	27	25
Other food	5.18	9.25	4.67	13	11	14
Wine	5.05	8.62	4.61	15	26	16
Pellet	4.49	8.13	4.04	22	28	23
Energy crops	5.71	7.70	5.46	8	33	5
Forestry	4.96	7.48	4.64	17	34	15
Bioelectricity	4.94	6.82	4.71	18	35	13
Biofuel—1st	4.07	9.35	3.41	29	9	29
Biofuel—2nd	4.45	8.96	3.88	24	18	26
Biochemicals	3.99	9.00	3.36	31	16	31
Textiles	3.13	8.98	2.39	33	17	33
Wood	5.02	8.94	4.53	16	19	18
Natural resources	2.37	9.15	1.52	35	12	35
Energy	5.36	8.69	4.94	11	24	11
Manufactures	4.08	9.01	3.46	28	15	28
Services	5.59	8.04	5.28	10	29	9

Source: own elaboration. Database: symmetric bioeconomy SAM for Spain 2010.
